# Anterior cruciate ligament reconstruction using quadriceps tendon autograft for adolescents with open physes- a technical note

**DOI:** 10.1186/1758-2555-3-7

**Published:** 2011-04-08

**Authors:** Christian Mauch, Markus P Arnold, André Wirries, Ralph R Mayer, Niklaus F Friederich, Michael T Hirschmann

**Affiliations:** 1 Department of Orthopaedic Surgery and Traumatology, Kantonsspital Bruderholz, Bruderholz, CH-4101, Switzerland; 2 Orthopaedic Surgery, Praxisklinik 2000, Freiburg, 79110, Germany

**Keywords:** transphyseal drilling, quadriceps tendon autograft, arthroscopy, ACL reconstruction, ACL tear

## Abstract

**Background:**

One major concern in the treatment of ACL lesions in children and adolescents with open physes is the risk of iatrogenic damage to the physes and a possibly resulting growth disturbance.

**Purpose:**

The primary purpose of this article is to describe our technique of a transphyseal ACL reconstruction using quadriceps tendon-bone autograft in children and adolescents with open growth plates. The secondary aim is to report our early results in terms of postoperative growth disturbances which are considered to be a major concern in this challenging group of patients. It was our hypothesis that with our proposed technique no significant growth disturbances would occur.

**Methods:**

From January 1997 to December 2007 49 consecutive children and adolescents with open growth plates were treated for a torn ACL using the aforementioned surgical technique. The patients (28 males and 21 females) with a median age at surgery of 13 (range 8-15) years were retrospectively evaluated. Outcome measures were follow-up radiographs (weight-bearing long leg radiographs of the injured and uninjured knee, anteroposterior and lateral views, a tangential view of the patella and a tunnel view of the injured knee) and follow-up notes (6 weeks, 3, 6, 12 months and until closing of physes) for occurrence of any tibial and/or femoral growth changes.

Results: All of the 49 patients had a sufficient clinical and radiological follow-up (minimum 5 years, rate 100%). 48 cases did not show any clinical and radiological growth disturbance. One case of growth disturbance in a 10.5 years old girl was observed. She developed a progressive valgus-flexion deformity which was attributed to a malplacement of the autograft bone block within the femoral posterolateral epiphyseal plate leading to an early localized growth stop. None of the patients were reoperated due to ACL graft failure. Five of the patients underwent revision ACL surgery due to another adequate sports trauma after the growth-stop. The tibial fixation screw had to be removed under local anaesthesia in 10 patients.

**Conclusions:**

The described ACL reconstruction technique represents a promising alternative to previously described procedures in the treatment of children and adolescents with open growth plates. Using quadriceps tendon future graft availability is not compromised, as the most frequently used autograft-source, ipsilateral hamstring tendons, remains untouched.

## Background

The incidence of midsubstance anterior cruciate ligament (ACL) tears in children and adolescents seems to increase over the last decades [1,2]. This can be explained at least partly by the fact that children more frequently and at younger age start to participate in high impact leisure and sport activities [3-5].

Nowadays most authors agree that early reconstructive surgery is able to prevent secondary meniscal and/or cartilage injuries, which tends to occur in the natural course of an unstable knee joint in this young and active patient's group [6-11].

Although a variety of surgical techniques have been reported, the optimal surgical method is still under debate [4].

One major concern in the treatment of ACL lesions in children and adolescents with open physes is the risk of iatrogenic damage to the physes and a possibly resulting growth disturbance [3,12-15]

Purely extraarticular techniques can be differentiated from physeal sparing and partial or complete transphyseal methods [4].

**Figure 1:**
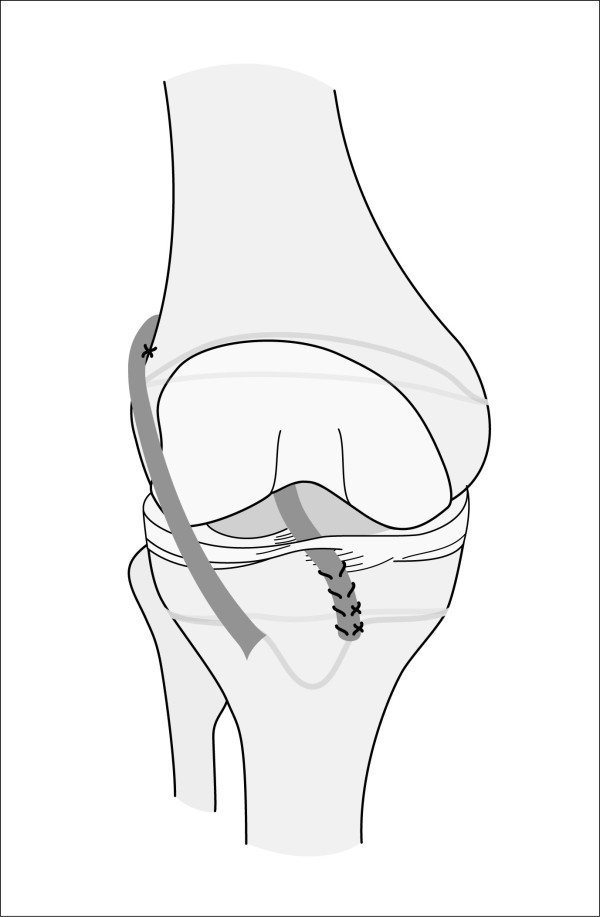
**Schematic drawing of an example of an extraarticular technique for ACL reconstruction in patients with open physes**.

Purely extraarticular techniques (Figure 1) such as the modified McIntosh & Darby technique frequently utilize a fascia-lata-stripe. One end stays attached to its origin at the Gerdy's tubercle and the other end is brought from extra- to intraarticular by twining it around the posterior part of the lateral femoral condyle [16-18].

**Figure 2:**
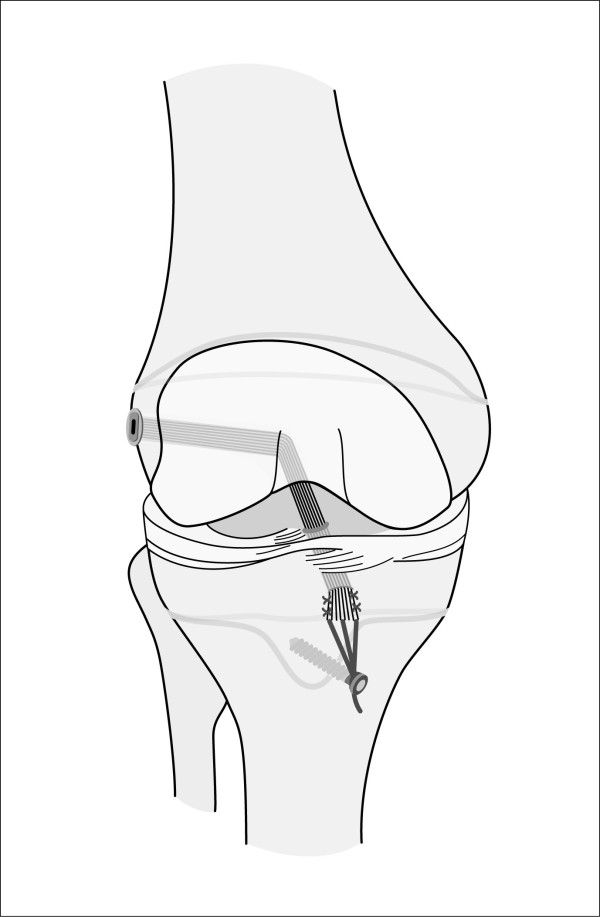
**Schematic drawing of an example of a physeal sparing ACL reconstruction technique in patients with open physes**.

Physeal sparing techniques (Figure 2) such as described by Anderson remain the physes untouched avoiding transphyseal tunnel drilling but are rarely reported [15,19].

Transphyseal techniques, establish their tibial and/or femoral tunnels by transphyseal drilling, and are either named as partial (only tibial) or complete (tibial and femoral) dependent whether all or only one physes is drilled through [4].

Chotel described a partial transphyseal technique using quadriceps tendon autograft being placed extraarticular under the lateral femoral condyle and attached intraarticular through a transphyseal tibial tunnel which leaves the femoral physis untouched [12,20].

The complete transphyseal ACL reconstruction does not differ significantly from techniques being used in adults. However, different tunnel starting points and angulations are recommended and the patellar tendon is less frequently used as graft [21-23].

Although there is a tremendous amount of scientific data on the treatment of ACL lesions in children and adolescents with open growth plates, the surgical method still seems to be more a matter of preference than evidence [24].

The purpose of this article is to describe the technique and report our early results of transphyseal ACL reconstruction using quadriceps tendon autograft in children and adolescents with open growth plates. Our hypothesis was that with our proposed technique no significant growth disturbances would occur.

## Material and methods

### Surgical technique (figure 3)

**Figure 3:**
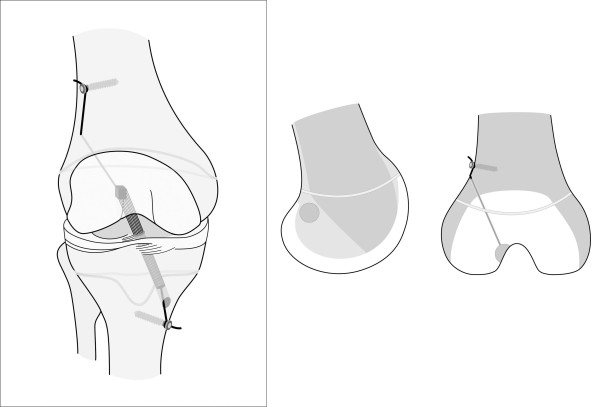
**Illustration showing the described transphyseal surgical ACL reconstruction technique using quadriceps tendon autograft in patients with open physes**.

In all patients an arthroscopically assisted anatomical single-bundle ACL reconstruction using an ipsilateral quadriceps tendon autograft (QT) was performed.

**Figure 4:**
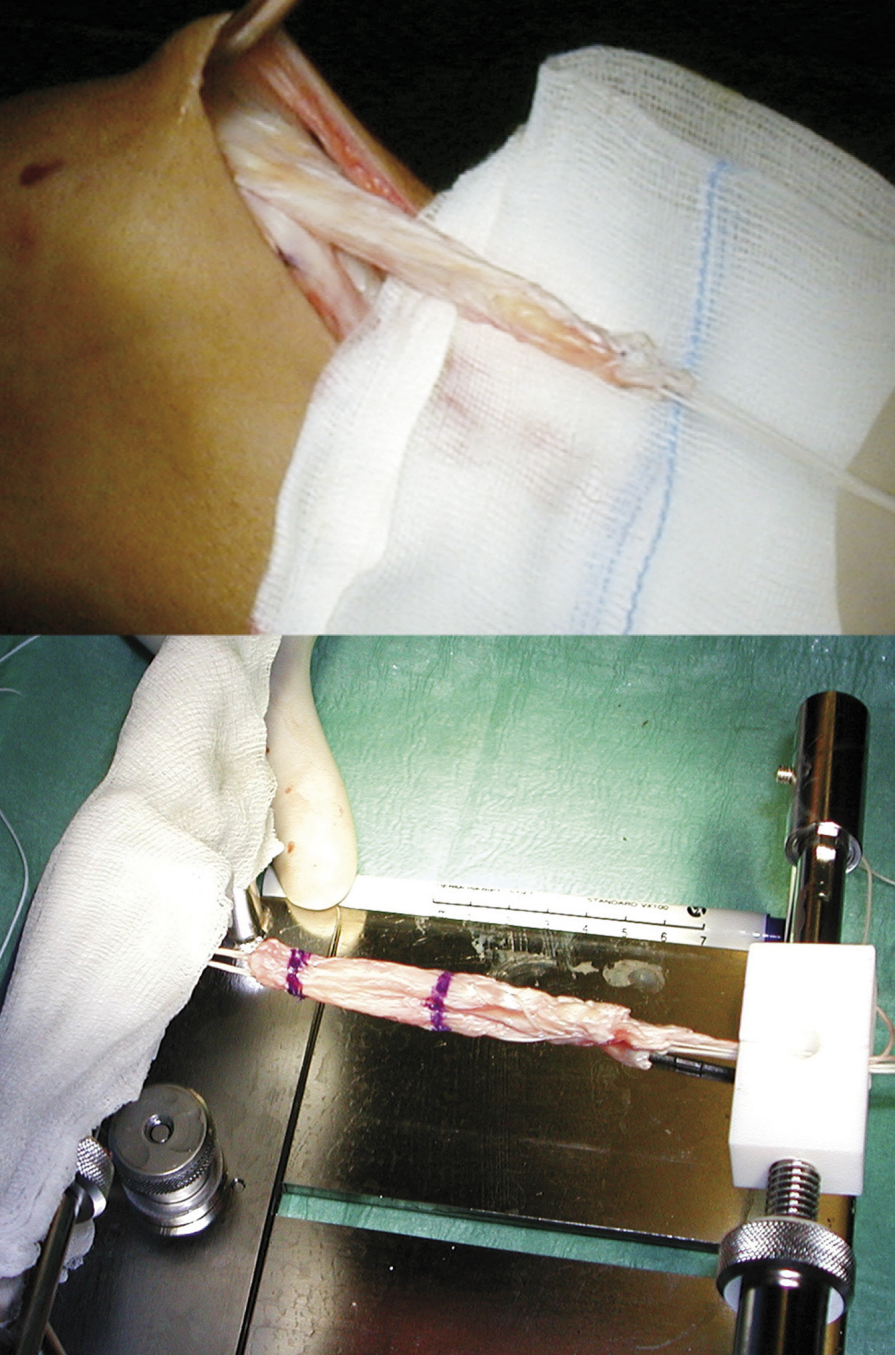
**A at least 7 cm long quadriceps tendon autograft is harvested, shaped and then armed with two non resorbable sutures on the bone and tendon end**.

Harvesting of the QT graft was done using a commercially available holding device with the knee between 90° of flexion and full extension to minimize the skin incision. A lateral suprapatellar skin incision of 3-4 cm length starting from the proximal patella pole was performed. The fascia and the tendon were bluntly separated. The QT was longitudinally incised starting proximally considering the various anatomical orientations of the QT (Figure 4).

Before harvesting the bone block 2.0 mm drill holes were made in the distal corners of the block in order to avoid fracturing of the patella while harvesting the graft. The preparation of the distal bone block (6-8 mm long and 5-6 mm wide) was finished using an 8 mm oscillating saw. The second cut in the QT was made parallel to the first one and 10 to 12 mm medially starting distally. The goal was to get a at least 7 cm long graft. Finally the proximal transverse cut of the QT was performed.

The bone block and the proximal QT were armed with two non resorbable sutures each (Synthofil 3, Braun, Melsungen, Germany). The harvest site was closed by suturing of the medial and lateral remaining QT (PDS 0, Johnson & Johnson, Spreitenbach, Switzerland). Then the fascia and the subcutaneous layer was closed in layers.

Arthroscopic part of surgery: A high anterolateral portal in the corner between the patella and the patella tendon was established with a vertical incision. A second, low anteromedial portal was performed under arthroscopic control. It was our aim to be as close as possible to the base of the anterior horn of the medial meniscus without harming it. A horizontal incision of the skin further helped to avoid lesions of the infrapatellar branches of the saphenous nerve. The intercondylar notch, tibial and femoral attachment areas were cleaned using a shaver blade.

**Figure 5:**
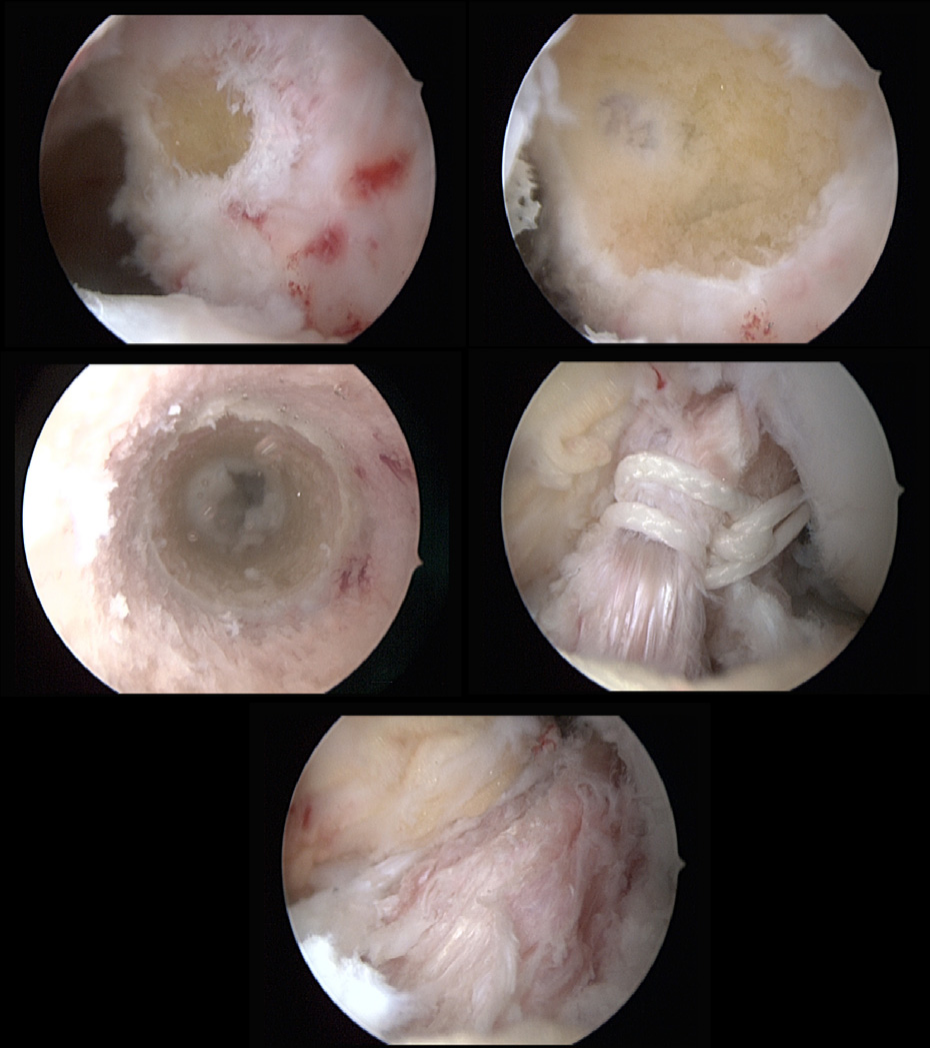
**Intraoperative arthroscopic images of the proposed transphyseal surgical ACL reconstruction technique using quadriceps tendon autograft in patients with open physes**. A The intercondylar notch and femoral attachment areas were cleaned using a shaver blade. A burr was used to create a groove in the very posterior aspect of the lateral side wall of the notch from 2:30 o'clock for a left and from 9:30 for a right knee respectively in order to stay distally to the physis. B The femoral position is found under standard arthroscopic view with the knee in 70 degrees of flexion using the high anterolateral portal. A groove is established just posterior to the resident ridge in the 2:30 or 9:30 o'clock position, a posterior wall of at least 3 mm should remain. in adults we go 1-2 mm furthermore posterior. After verification of the correct tunnel position via the medial portal the groove was gradually deepened using 6-8 mm wide surgical spoons. The 8 mm spoon should completely fit into the groove simulating the future position of the bone block. During preparation of the groove it was mandatory to meticulously control the posterior cortical wall to prevent iatrogenic posterior wall break out. C Tibial intra-tunnel view demonstrating its small diameter. The starting point of the tibial tunnel was medially and distally compared to the standard adult technique to pass the physis in an steep angle and as far central as possible to harm as less as possible of the physis and to spare the tibial apophysis. D Retrograde introduction of the graft in the femoral bone groove. E Image after insertion of the quadriceps tendon autograft at the end of surgery.

A burr was used to create a groove in the very posterior aspect of the lateral side wall of the notch from 2:30 o'clock for a left and from 9:30 for a right knee respectively in order to stay distally to the physis (Figure 5A). The femoral position is found under standard arthroscopic view with the knee in 70 degrees of flexion using the high anterolateral portal. A groove is established just posterior to the resident ridge in the 2:30 or 9:30 o'clock position, a posterior wall of at least 3 mm should remain. in adults we go 1-2 mm furthermore posterior.

This was made in a slightly different position than in adults in order not to damage the femoral physis (Figure 3). After verification of the correct tunnel position via the medial portal the groove was gradually deepened using 6-8 mm wide surgical spoons. The 8 mm spoon should completely fit into the groove simulating the future position of the bone block. During preparation of the groove it was mandatory to meticulously control the posterior cortical wall to prevent iatrogenic posterior wall break out (Figure 5A).

A second incision of 3 cm was performed starting approximately 4 cm proximal to the lateral condyle. The iliotibial tract was split longitudinally anterior to the intermuscular septum. A hook was placed under the lateral vastus muscle and bluntly elevated. A 3.5 mm post screw (modified cortical screw with long neck, Synthes, Oberdorf, Switzerland) was placed into the distal femur. The femoral tunnel was drilled via a 4.5 mm cannula using the AM tunnel in 120 degrees of knee flexion starting in the posterolateral bottom of the groove over the top aiming towards the surgeon's index finger of the 2nd hand on the lateral cortex of the femur, using a 3.5 mm drill. A shuttle was then passed through for later graft passage.

A third longitudinal skin incision of 2 cm was performed medial and distal to the tibial tuberosity. The starting point of the tibial tunnel was medially and distally compared to the standard adult technique to pass the physis in an steep angle and as far central as possible to harm as less as possible of the physis and to spare the tibial apophysis (Figure 5B). The shuttle was then passed out tibially and the QT graft was inserted in retrograde direction (Figure 5C+D). The bone block was press fit into the groove under arthroscopic control. A firm endpoint was regularly noted. Then the proximal sutures were knotted around the femoral post screw. The distal sutures were fixed in 10°-15° of knee flexion after conditioning of the graft in 10 cycles on a second tibial post screw.

### Postoperative treatment

An early functional rehabilitation program with passive range of motion training, electrical muscle stimulation and closed chain quadriceps and hamstring exercises was initiated. For a maximum of two weeks ambulation with full weight bearing was only allowed in full extension. Passive range of motion on a continuous passive motion machine was initiated on day one after surgery. During 8 weeks the patient was mobilised in an extension brace.

Sports activity was initiated 6 months postoperatively, cutting and pivoting sport nine months postoperatively.

### Follow-up

From January 1997 to December 2007 49 consecutive children and adolescents with open growth plates were treated for a torn ACL using the aforementioned surgical technique. Twenty of these patients were isolated ACL injuries, 29 had an associated meniscal injury and in 9 cartilage lesions >grade II ICRS were present. In 21 patients a meniscal suture and in 8 a partial meniscectomy was performed.

The patients (28 males and 21 females) with a median age at surgery of 13 (range 8-16) years were retrospectively evaluated on follow-up radiographs (weight-bearing long leg radiographs of the injured and uninjured knee, anteroposterior and lateral views, a tangential view of the patella and a tunnel view of the injured knee). and follow-up notes (6 weeks, 3, 6, 12 months and until closing of physes) for occurrence of any tibial and/or femoral growth changes.

## Results

**Figure 6:**
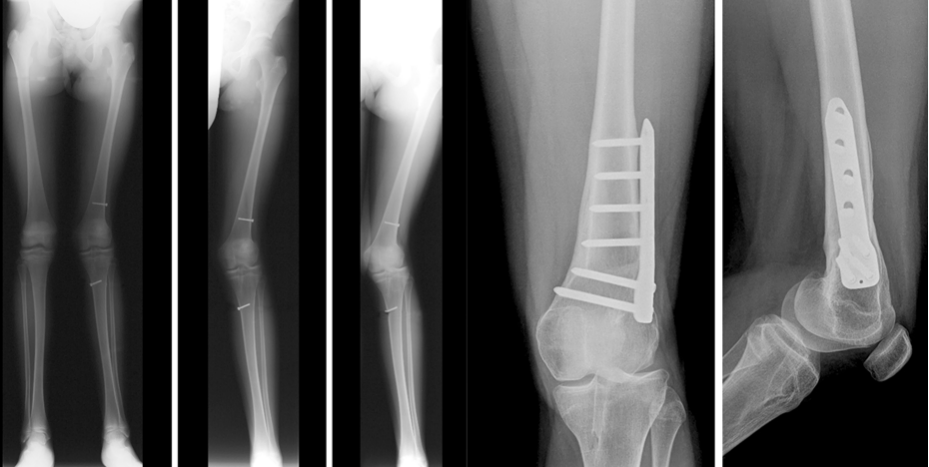
**Progressive valgus and flexion deformity of a patient after QT ACL reconstruction and after distal femoral varisation osteotomy**.

**Figure 7:**
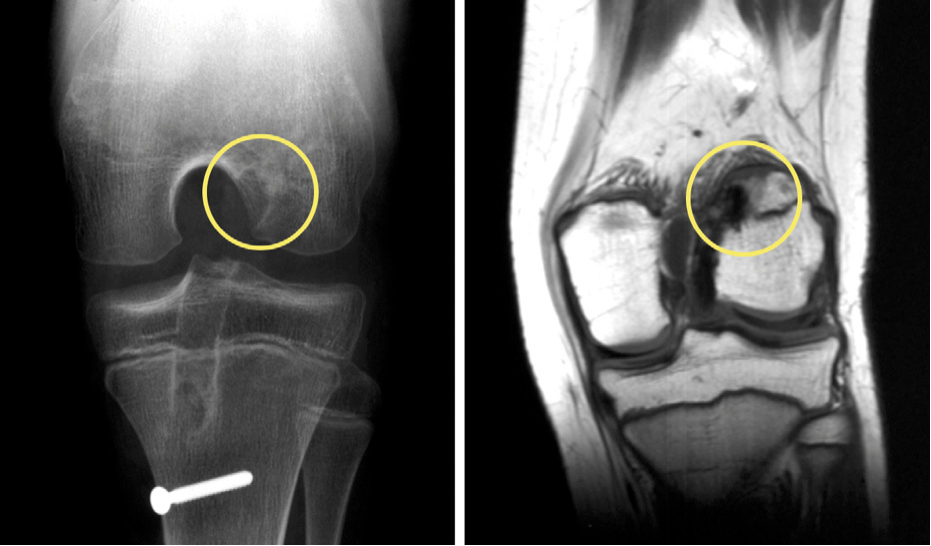
**The early localized growth stop was attributed to a malplacement of the autograft bone block within the femoral posterolateral epiphyseal plate**. The femoral bone block was placed to high in the notch damaging the femoral growth plate.

All of the 49 patients had a sufficient full clinical and radiological follow-up (follow-up minimum 5 years, rate 100%). 48 cases did not show any clinical and radiological growth disturbance. There was one exception. Clinically and radiologically one case of growth disturbance in a girl that was aged 10.5 years by the time of operation was observed (Figure 6). She developed a progressive valgus-flexion deformity which was attributed to a malplacement of the autograft bone block within the femoral posterolateral epiphyseal plate leading to an early localized growth stop (Figure 7). After closing of the physes the valgus deformity was corrected using a supracondylar varisation-extension-osteotomy. The recovery was uneventful and the patient was pain free at last follow-up 12 months after revision surgery. She returned to sport 6 months after osteotomy.

None of the patients were reoperated due to ACL graft failure within the follow up period of maximum 7 years. Five of the patients underwent revision ACL surgery due to another adequate sports trauma after the growth-stop. The tibial fixation screw had to be removed under local anaesthesia in 10 patients.

## Discussion

A variety of different arthroscopic surgical methods have been reported [4,24]. This high number of different methods reflects the fact, that there still is no consensus on how to treat ACL injuries in children and adolescents and avoid growth disturbances [25,26].

The described technique offers the following major advantages.

Firstly, the reported technique for ACL reconstruction in children and adolescents with open physes is safe, simple and as anatomical as possible. Although the technique includes transphyseal drilling for the femoral and tibial side only one case of significant clinically significant growth disturbance was observed, which we attribute to the fact that the transphyseal bone tunnels are small in diameter and steep angled. In addition, only the suture (femoral) or the suture and the graft (tibial) are in contact with the physes. The bone block itself has no contact to the physes, which minimizes the risk of physeal ossification and subsequent growth disturbance [13,27]. From animal studies it was shown that the angle of transphyseal drilling and the type of graft material significantly influences ossification of the physes leading to partial arrest of the growth and significant bone deformity [13,14,21,27]. A growth arrest is more likely to occur when more than 3-5% of the physis diameter is damaged, whereas a soft tissue graft within the tunnel seems to reduce the probability [13,14,28,29].

According to Kocher et al. growth disturbances predominantly occur at the femoral physis, which is in accordance with our results [3,12,27,30]. In a survey of the Herodicus Society and ACL Study Group a valgus deformity of the femur appeared to be the most frequent complication in this challenging group of patients [3].

The second most common growth disturbance was a genu recurvatum caused by damage of the tibial apophysis during tibial fixation of the autograft [3]. Using our surgical technique with QT autograft the damage of the tibial apophysis is avoided.

Interestingly, even in procedures sparing the growth plates, growths disturbances were described, which might be explained by excessive pulling of the implanted autograft then leading to indirect growth deceleration [31]. However, the exact pathological process remains unclear. The described case of growth disturbance after ACL reconstruction using our proposed technique could be attributed to a malposition of the femoral tunnel position. The femoral attachment was placed too high in the notch and damaged the femoral physis.

Secondly, using an ipsilateral quadriceps tendon bone autograft combines the advantage of immediate femoral press-fit fixation with the strong biomechanical characteristics of the QT, which is comparable to a patella tendon autograft [32-34]. The integration of the graft is facilitated by direct contact of the bone block and the prepared groove. Along with the press-fit fixation the femoral graft attachment, which is naturally defined by the tendon-bone block interface, optimally mimics the anatomical attachment [35,36]. We believe that reducing the autograft turning at insertion site offers a significant benefit of our technique as it is theoretically able to reduce the shearing and windshield wiping forces [36,37].

Furthermore, children typically do not show enough insight and compliance in the postoperative rehabilitation, which in combination with the use of the ipsilateral QT autograft and its related postoperative pain and quadriceps inhibition act as an inherent break to overstressing the graft complex postoperatively.

In addition, another strong argument for use of the QT autograft is the fact that the hamstring tendons remain untouched. This might be particularly helpful in children and adolescents, in which in future additional reconstructions have to be performed and only a limited number of autografts is available. To date, it is also questionable if it is advisable to use two of the three internal knee rotators as graft source and what the mid-term consequences will be [38].

The following limitations have to mentioned. There were no clinical or radiological outcome scores reported as this was not the intention of the report. However, to give evidence that the described technique results in good clinical outcome a further prospective study should focus on clinical outcome of patients treated with this technique.

## Conclusions

The described ACL reconstruction technique represents a promising alternative to previously described procedures in the treatment of children and adolescents with open growth plates. As shown it does not lead to significant growth disturbances. In addition, using the quadriceps tendon it does not compromise later graft availability, as the most frequently used autograft, ipsilateral hamstring tendons, remains untouched.

## Note

Written informed consent was obtained from the patient for publication of this case report and accompanying images. A copy of the written consent is available for review by the Editor-in-Chief of this journal.
